# Quantum causality emerging in a delayed-choice quantum Cheshire Cat experiment with neutrons

**DOI:** 10.1038/s41598-023-29970-6

**Published:** 2023-03-08

**Authors:** Richard Wagner, Wenzel Kersten, Hartmut Lemmel, Stephan Sponar, Yuji Hasegawa

**Affiliations:** 1 Atominstitut, TU Wien, Stadionallee 2, 1020 Vienna, Austria; 2 Institut Laue Langevin, 38000 Grenoble, France; 3 Department of Applied Physics, Hokkaido University, Kita-ku, Sapporo 060-8628 Japan

**Keywords:** Quantum physics, Matter waves and particle beams

## Abstract

We report an experiment with neutrons in a silicon perfect crystal interferometer, that realizes a quantum Cheshire Cat in a delayed choice setting. In our setup the quantum Cheshire Cat is established by spatially separating the particle and its property (i.e. the neutron and its spin) into the two different paths of the interferometer. The condition for a delayed choice setting is achieved by postponing the choice of path assignment for the quantum Cheshire Cat, i.e. which path is taken by the particle and which by its property, until the point in time when the neutron wave function has already split and entered the interferometer. The results of the experiment suggest not only the fact that the neutrons and its spin are separated and take different paths in the interferometer, but also quantum-mechanical causality is implied, insomuch that the behavior of a quantum system is affected by the choice of the selection at a later point in time.

## Introduction

The 20th century made available to us the deeply mysterious yet profoundly accurate and broad ranging formalism of quantum mechanics that provides an astounding number of quantitatively correct predictions^1,2^ and yet shows counter-intuitive and paradox peculiarities^3^ that puzzle until today. An important aspect of the theory is the wave-particle dualism^4^ that has often been demonstrated in Mach-Zehnder interferometer experiments (see e.g.^5^, an experimental study on single-photon level). The interference pattern(fringes) and the information which path a particle inside an interferometer took, are quantum mechanical complementary quantities^6,7^. If the paths are known the interference fringes vanish and vice versa, if we observe interference fringes we have no information, which path the particle took. To show how this may lead to counter-intuitive behavior, Wheeler put forward his delayed-choice gedanken experiment^8,9^. He proposed a setup where the influence of the choice, whether to observe the wave property (interference) or the particle property (path) is delayed until the photons had already split into the two paths in the interferometer. In this gedanken experiment, particles passing the first beam splitter do not know whether they should behave as a wave or a particle, i.e., to take one or both paths. Putting a second beam splitter in place (or removing it) at the exit of the interferometer, *after* the particle has already entered it, can have an effect on its past history. This consideration may suggest that, contrary to what common sense would suggest, the quantum feature of a photon, i.e, showing the wave or particle property, depends solely on the (delayed-) choice of the experimenter, that actually takes place at a time subsequent to a reference time of the photon splitting. This led Wheeler to conclude “We decide what the photon* shall have done* after it has *already* done it”^10^. Another of his often cited dictums in this matter is: “The past exists only insofar as it is recorded in the present” (as quoted in^11^). Several such experiments concerning the wave-particle dualism^12–14^ as well as quantum swapping^15^ and quantum eraser^16^ with a delayed-choice option are reported. A very recent example^17^ studies the quantum superposition of wave-particle states in a non-local setup of a delayed-choice experiment. It was argued that one of the simplest but counter-intuitive analyses would be to allow influences from the future to the past, so that influences travel backwards in time, constituting an effect of retro-causality^18,19^. This constitutes an example of quantum causality, where, in contrast to classical causality, the causal order of events is indefinite^20,21^. We want to note that another way to explain a delayed choice experiment is offered in the realistic interpretation of quantum mechanics (REIN)^22^. The wave function is taken to really exist so a quantum object can be present in disjointed regions of space and collapse^23^ instantly upon a measurement. In this interpretation, wave- or particle-like nature in a delayed-choice Mach-Zehnder interferometer is assigned to the observed interference or non-interference.

A Quantum Cheshire Cat (qCC), named after its counterpart in Lewis Carroll’s famous novel “Alice’s Adventures in Wonderland”^24^, is a quantum mechanical phenomenon put forward by Yakir Aharonov et al.^25^. It describes the paradoxical situation for a quantum system that is prepared in a certain state (pre-selection) and evolved into another specific state (post-selection), when it is suggested that a quantum object (“the cat”) is separated from its property (“the grin”). The first experimental demonstration of the qCC has been reported for a silicon perfect crystal interferometer setup with neutrons^26^ and was followed up by one using a photonic system^27^. The significance and consequences of such a disembodiment, including its philosophical implications and its interpretation, raised discussions and speculations^28–31^, whether the effect is real or whether actual proof of it has been achieved. A number of theoretical proposals for further investigation in the qCC effect have been put forward recently^32–35^ and in recent experiments with entangled photon pairs^36^ the exchange of localized properties of a qCC and a scheme of noninvasive weak measurement^37^ have been shown. Further we would like to point out that another new theoretical concept of a dynamical qCC where a flux of disembodiment in space is constituted has been introduced lately^38^.

In the early demonstration of a quantum Cheshire Cat^26^ with neutrons, one finds the particle and its property spatially separated from each other and located in the different paths of the interferometer. Theory predicts that the separation and the location of the particle and its property depend solely on the post-selected final state. In such a setup the following questions arise: what happens if the decision of the choice of the post-selection is postponed until the quantum particle had already entered the interferometer? Will the particle and its property still be separated spatially? Or to make the question even more pointed: can the location of the separated particle and its property be influenced by the choice of the post-selection, that has been decided upon *afterwards*?

**Figure 1:**
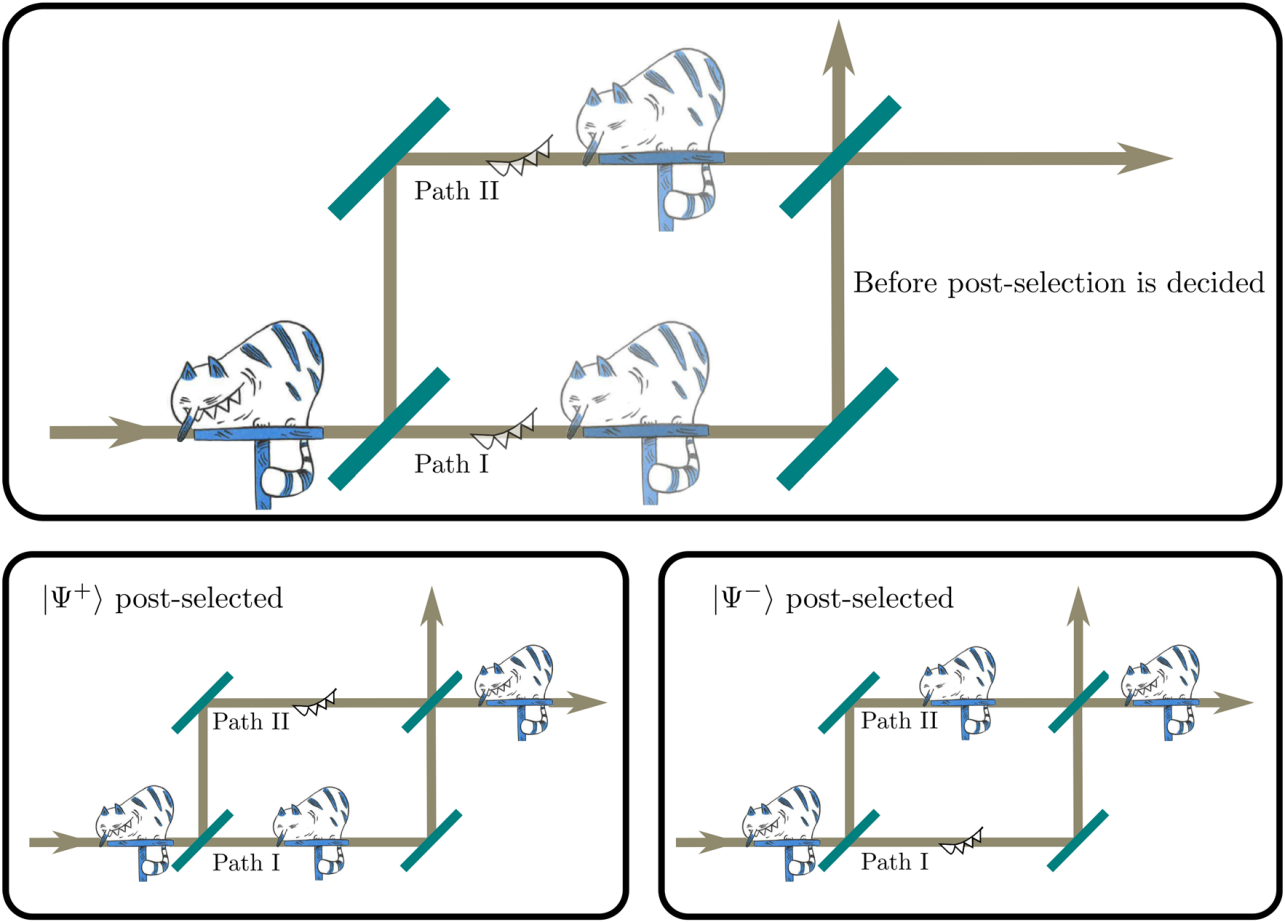
Artistic sketch of the delayed choice quantum Cheshire Cat Experiment. Top: After the cat enters the interferometer the position of body and grin is undecided. Bottom left: If ${|{\Psi ^{+}}\rangle }$ is post-selected, the body took the lower and the grin the upper path. Bottom right: If ${|{\Psi ^{-}}\rangle }$ is post-selected, the behavior is exactly the opposite. Cat courtesy of Nicolas Mahler^39^.

In the present work, we apply a fast switch in the choice of the post-selection to the set up of the above mentioned qCC experiment^26^. The switching is done so fast that neutrons in the split beams of the interferometer do not know which post-selection will be applied, i.e. where the particles and its properties should be located, until they already started propagating through the interferometer. In Fig. 1 an artistic depiction of the experiment is shown.

The type of qCC that emanates in the interferometer is dependent on the delayed choice, since according to quantum theory the location of the neutrons and its properties are determined by the post-selection chosen and applied at the time yet to come; the neutron’s behavior in the interferometer is governed solely by the action at a subsequent time. The observed outcomes of the experiment, by quickly switching the choice of the post-selection, can such be attributed to quantum causality.

## Results

### Theoretical framework for the experiment

The essential elements of the present qCC experiment are the (pre-selected) initial state ${|{\Psi _i}\rangle }$ and the (post-selected) final states ${|{\Psi _f}\rangle }$. They are written in the form

1 $$\begin{aligned} \left\{ { \begin{array}{*{20}l} {|{\Psi _i}\rangle } = \dfrac{1}{\sqrt{2}}({|{+x}\rangle }{|{\text {I}}\rangle } + {|{-x}\rangle }{|{\text {II}}\rangle }) \\ {|{\Psi ^{\pm }_f}\rangle } = \dfrac{1}{\sqrt{2}}{|{\pm x}\rangle }({|{\text {I}}\rangle } + {|{\text {II}}\rangle }), \end{array} } \right. \end{aligned}$$

where ${|{\pm x}\rangle }$ denotes the direction of the spin analysis and ${|{{\text {I}}}\rangle }$ and ${|{{\text {II}}}\rangle }$, the path I and the path II in the interferometer. The superscript in the post-selected state (±) represents the two different choices of the spin analysis, which will be switched dynamically in the experiment. Weak values, as introduced by Aharonov, Albert and Vaidman^40^ in course of their examination of the quantum measurement process, are used to evaluate the dynamical behavior of the above states in the interferometer. For an observable ${\hat{A}}$ of a system, the weak value is defined as follows:

2 $$\begin{aligned} {\langle {{\hat{A}}}\rangle }_w = \frac{{\langle {\Psi _f}|}{\hat{A}}{|{\Psi _i}\rangle }}{{\langle {\Psi _f|\Psi _i}\rangle }}. \end{aligned}$$

With ${|{\Psi _i}\rangle }$ being the initial, or pre-selected, state and ${|{\Psi _f}\rangle }$ the final, or post-selected, state of the system under observation. This definition is now applied to the states given by Eq.(1) and the following observables: Firstly to the projection operators of each interferometer path ${\hat{\Pi }}_{\text {I}} = {|{\text {I}}\rangle }{\langle {\text {I}}|}$ and ${\hat{\Pi }}_{\text {II}} = {|{\text {II}}\rangle }{\langle {\text {II}}|}$. Secondly to the spin operator conditioned on each path $\hat{\sigma _z}{\hat{\Pi }}_{\text {j}}$ with $j=\{\text {I},\text {II}\}$, where $\hat{\sigma _z}$ is the Pauli spin operator, hence representing the observable of the neutron’s spin z-component in the path *j*. For the defined initial and final states we get the result:

3a $$\begin{aligned} \left\{ { \begin{array}{*{20}l} \langle {\hat{\Pi }}_{\text {I}} \rangle ^+_w = 1, \langle {\hat{\Pi }}_{\text {II}} \rangle ^+_w = 0 \\ \langle {\hat{\sigma }}_z{\hat{\Pi }}_{\text {I}} \rangle ^+_w = 0, \langle {\hat{\sigma }}_z{\hat{\Pi }}_{\text {II}} \rangle ^+_w = 1 \end{array} \text { for the final state} {|{\Psi ^+_f}\rangle } } \right. \end{aligned}$$

and

3b $$\begin{aligned} \left\{ { \begin{array}{*{20}l} \langle {\hat{\Pi }}_{\text {I}} \rangle ^-_w = 0, \langle {\hat{\Pi }}_{\text {II}} \rangle ^-_w = 1 \\ \langle \hat{\sigma _z}{\hat{\Pi }}_{\text {I}} \rangle ^-_w = 1, \langle \hat{\sigma _z}{\hat{\Pi }}_{\text {II}} \rangle ^-_w = 0 \end{array} \text { for the final state} {|{\Psi ^-_f}\rangle } } \right. \end{aligned}$$

The Eqs. (3a) and (3b) suggest two important features of the location of neutrons and the spin by switching the choice of the post-selection: (i) The first lines indicate that the neutrons are found to be localized in different paths by switching the choice of the post-selection; they are found in the path I and II by applying the post-selection ${|{\Psi ^{+}_f}\rangle }$ and ${|{\Psi ^{-}_f}\rangle }$, respectively. (ii) The lines of the second part of the equations indicate that the spin in the different paths is found to be affected by switching the choice of the post-selection; the spin in path II and I is affected by applying the post-selection ${|{\Psi ^{+}_f}\rangle }$ and ${|{\Psi ^{-}_f}\rangle }$, respectively. Note that, in both choices of the post-selection, neutron and spin are localized in different paths, i.e., the location of the cat itself and its grin are interchanged by switching the choices of the post-selection. Since measurement of the locations of the neutron and the spin in the interferometer can be carried out independently of the delayed-choice process, the picking of a direction for post-selection, the influence of the delayed-choice on the preceding measurements can be investigated. We would like to point out that the experimental proposal in a recent publication^35^, contains a delayed choice scenario, too. The difference to the experiment presented in this report is that the authors of^35^ suggest a setup where two properties of the same system, represented by two non-commuting observables, are separated. In contrast to that, we deal in our experiment with the separation of one property from the system itself, hereby constituting the phenomenon of disembodiment. Further we would like to point out that in their Gedanken-experiment the effect of a change in the pre-selection is discussed that in our view has no retro-causal implications.

### Experiment

**Figure 2:**
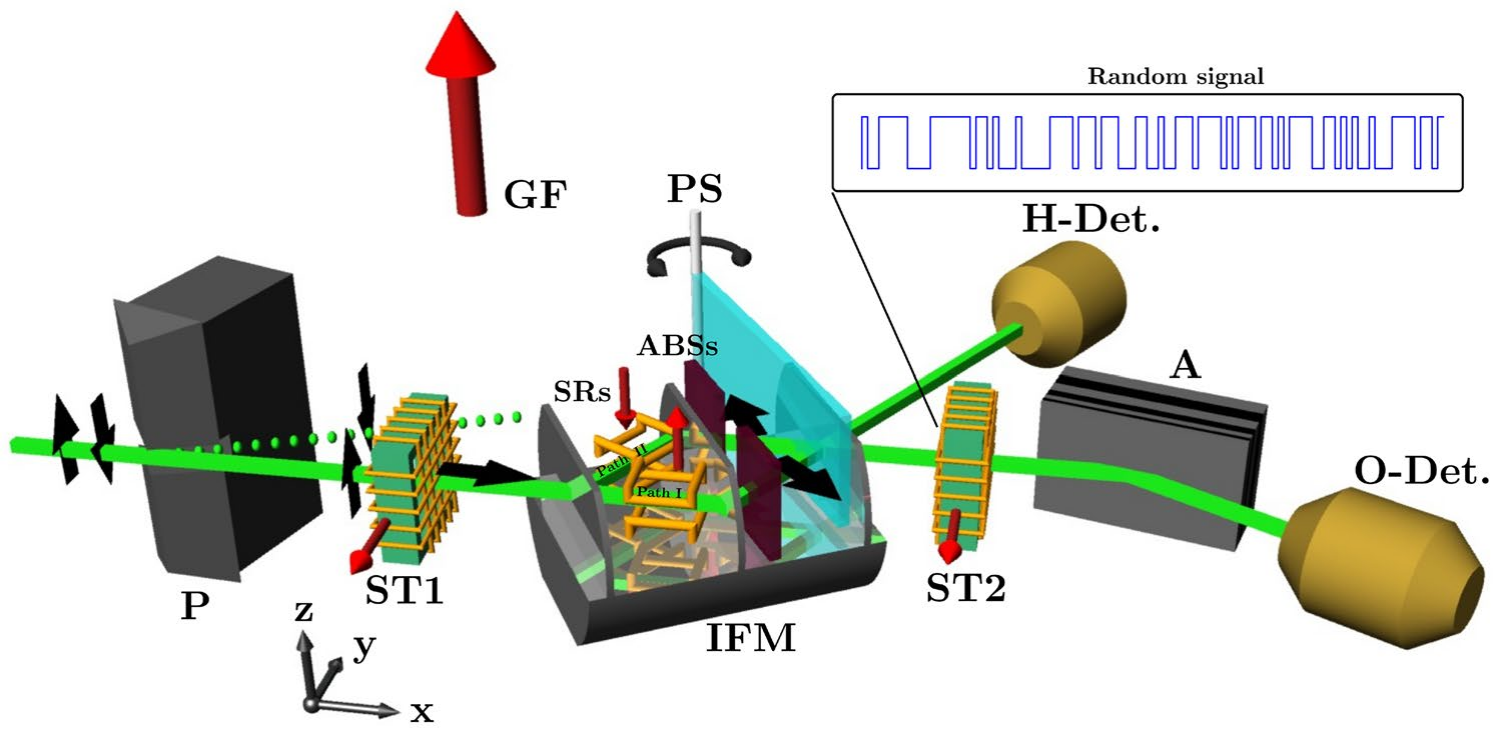
Experimental setup of the demonstration of quantum Cheshire Cat with delayed-choice option. The monochromatic neutron beam passes through magnetic birefringent prisms (P), thus the beam is polarized. To avoid unwanted depolarization of this beam, a fairly homogeneous magnetic guide field (GF) in the +z direction is applied around the whole setup. The spin turner 1 (ST1) before the interferometer rotates the neutron spin by $\pi /2$ into the $+x$ direction. Then the incident beam falls on the interferometer (IFM) and is split into two beams at the first plate of the IFM. Between the first and the second plate of the IFM, the neutron’s wave function is prepared in the state ${|{\Psi _i}\rangle } = \dfrac{1}{\sqrt{2}}({|{+x}\rangle }{|{\text {I}}\rangle } + {|{-x}\rangle }{|{\text {II}}\rangle })$ via rotation of the neutron’s spin with the two spin rotators (SRs). (Note that the guide field induces the additional spin rotation along the *z*-axis.) By applying small, additional spin-rotations with these SRs, the weak values of $\left\langle {\hat{\sigma }}_z{\hat{\Pi }}_{\text {I}}\right\rangle ^\pm _{\mathrm{w}}$ and $\left\langle {\hat{\sigma }}_z{\hat{\Pi }}_{\text {II}}\right\rangle ^\pm _{\mathrm{w}}$ are determined. The absorbers (ABSs) are inserted one by one at a time in the beam paths during the measurements of $\left\langle {\hat{\Pi }}_{\text {I}}\right\rangle ^\pm _{\mathrm{w}}$ and $\left\langle {\hat{\Pi }}_{\text {II}}\right\rangle ^\pm _{\mathrm{w}}$. The relative phase $\chi$ between the beams in paths I and II is adjusted by a phase shifter (PS). The two interfering beams from the interferometer in the forward and the reflected directions are monitored by the *O*- and *H*-detector, respectively. The spin of the interfering beam in the forward direction is analysed by the use of spin analyzer (A) together with a spin turner 2 (ST2), which fulfills the condition of post-selection of the states ${|{\Psi ^{\pm }_f}\rangle } = \dfrac{1}{\sqrt{2}}{|{\pm x}\rangle }({|{\text {I}}\rangle } + {|{\text {II}}\rangle })$ . Here, two voltage levels on the ST2 are switched quickly and at random so that neutrons in the interferometer do not know the choice of the post-selection, either ${|{\Psi ^+_f}\rangle }$ or ${|{\Psi ^-_f}\rangle }$.

The experiment was carried out at the S18 silicon-perfect-crystal interferometer beam line at the high flux reactor at the Institute Laue Langevin. A schematic view of the experimental set-up is shown in Fig. 2.

A monochromatic neutron beam with a wavelength of $\lambda = 1.92$ Å passes magnetic birefringent prisms, which polarize the beam of incoming neutrons vertically, and falls on a triple-Laue interferometer. Only the neutrons polarized in up ($+z$) direction enter the neutron interferometer at the correct angle. A fairly uniform magnetic guide field is produced over the setup by a pair of water-cooled Helmholtz coils, avoiding a loss of polarization of the associated neutron beams. The beam cross section is reduced to $5\times 4\,\hbox {mm}$ by a diaphragm before the interferometer. A spin turner is placed just before the interferometer, which turns the spin of the incoming neutron into the +x direction. At the entry of the triple-Laue interferometer the beam is split into two paths. A DC spin rotator in each one of them applies a $\pm \pi /2$-operation, and thus generates the initial state ${|{\Psi _i}\rangle }$, given by Eq. (1). A parallel-sided sapphire plate of ${3}\,\hbox {mm}$ thickness is used as a phase shifter to tune the relative phase $\chi$ between path $\text {I}$ and $\text {II}$. At the last plate of the interferometer, the state is projected into the forward direction (denoted *O*-beam) $\Psi _f = \dfrac{1}{\sqrt{2}}({|{\text {I}}\rangle } + \mathrm{e}^{\mathrm{i}\chi } {|{\text {II}}\rangle })$. The spin of this beam is analyzed with the help of a spin turner and a spin analyzer made of a polarizing supermirror. This spin analysis of the *O*-beam allows the post-selection of the final states ${|{\Psi ^\pm _f}\rangle }$ as in Eq. (1). A pencil-sized detector, 6 mm in diameter, filled with $^{3}$He is placed directly at the outgoing window of the polarizing supermirror. The time resolution of this detector is ${3}\,{\upmu \hbox {s}}$. This allows to resolve and record the arriving times of the neutrons and relate them to the switching state of the spin-analysis. No time resolved measurement is needed for the *H*-beam, the interfering beam in the reflected direction. Its intensity is recorded with a large shielded $^{3}$He counting tube, 50 mm in diameter and used as a monitor and reference for the setup.

#### Delayed-choice option

In contrast to a stationary spin-analysis that stays the same over the duration of the measurement, in the present experiment the direction of the spin-analysis is switched so fast that neutrons at the entrance of the interferometer do not know which post-selections will be realized afterwards. In cases of spin analysis other than in the directions $\pm x$, theory predicts no separation of the neutrons and its spin. Therefore, we decided to apply exactly these two directions (corresponding to the spin states ${|{+x}\rangle }$ and ${|{-x}\rangle }$), thus accomplishing the post-selections of the final states ${|{\Psi ^\pm _f}\rangle }$. The delayed-choice decision whether to realize the ${|{\Psi ^{+}_f}\rangle }$ or ${|{\Psi ^{-}_f}\rangle }$ post-selection is varied at random with ${100}\,{\upmu \hbox {s}}$ interval. This is shorter than the propagation time of neutrons (having velocity $v \approx {2}\,\hbox {km/s}$) in the present experiment, passing through the distance of ${35}\,\hbox {cm}$ from the first plate of the interferometer to the spin-turner used to switch the choice of the post-selection. We want to note that, although the interferometer and the device to randomly select a choice of post selection are spacelike separated, our experiment will also include timelike separated events (i.e. neutron enters the interferometer and a switching decision is made). From point of view of the neutron the point in time the switching decision is made is always after the point in time it entered the interferometer and no information is transferred from the random decision process to the neutron. The arrival times of the neutrons in the *O*-beam are recorded and they are subsequently classified according to the state of the delayed-choice post-selection. A detailed description of the fast switching together with the discrimination of neutrons according to their arriving times is given in the *Methods* section.

### Measurements of the locations of the neutrons and its spin component

Since the neutrons should be generated in the initial state ${|{\Psi _i}\rangle }$ and afterwards affected by the post-selection of the final states ${|{\Psi ^\pm _f}\rangle }$, the conventional method of quantum measurement, which is inevitable accompanied with certain recoils, cannot be applied for the measurements of the location in the interferometer. As suggested by theoreticians^25^ and realized in the previous experiment^26^, applying a weak interaction^40,41^ is expected to minimize such recoils and to ensure the accomplishment of the pre- and the post-selection. We follow this scheme by using a weak absorber or a weak interacting magnetic field, that are applied in each beams path in the interferometer, to evaluate the neutrons’ population and the spin’s position.

**Figure 3:**
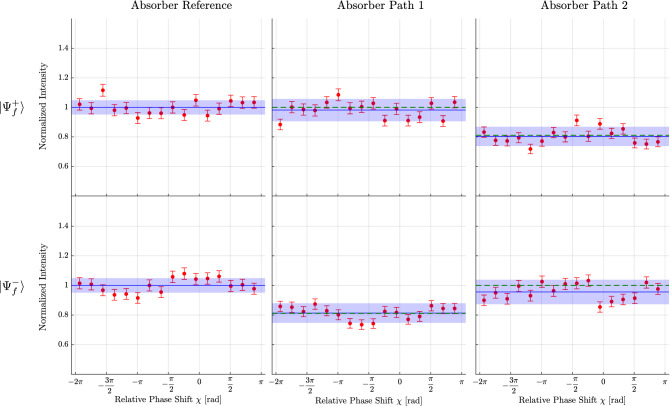
Typical result of the measurement of the neutrons’ location Interferograms are recorded by tuning the relative phase $\chi$ with the phase shifter. Three plots, one reference without the absorber and two with the absorber in the paths I/II, are obtained, each for the choices of the final states ${|{\Psi ^+_f}\rangle }$ and ${|{\Psi ^-_f}\rangle }$. The transmissivity of the absorber is $T=0.82(1)$ and the intensity is plotted as a function of the relative phase $\chi$. The solid lines are least-square fits to the data. Error bars and the filled area represent one standard deviation. Dashed lines indicate the theoretical prediction. In comparison with the reference measurement on the left column, two features are clearly seen. (1) For the post-selection of the state ${|{\Psi ^+_f}\rangle }$, the absorber put in path I leaves the intensity unchanged while in case it is put in path II the intensity is decreased; the neutrons propagate through the interferometer taking only path II. (2) For the post-selection of the state ${|{\Psi ^-_f}\rangle }$, the absorber in path I causes a decrease in the intensity and in path II leaves the intensity unchanged; the neutrons propagate through the interferometer taking only path I. Thus switching the states of the post-selection, the location of the neutrons is swapped between path I and path II.

At first the neutrons’ population is evaluated by the use of the weak absorber, made of an Indium plate of ${0.25}\,\hbox {mm}$ in thickness with the transmission $T=0.82$. This absorber plate is put in each of the beams inside the interferometer and interferograms are recorded by tuning the relative phase $\chi$. A separate measurement without the absorber serves as the reference. The fast switching of the spin-analysis after the interferometer is turned on for all the measurements. Six interferograms were obtained for the absorber measurement. Consisting of a set of three, one without the absorber and one with the absorber in the path I respectively path II, for each of the spin-analysis directions ${|{+x}\rangle }$ or ${|{-x}\rangle }$. The interferograms of the reference measurements are used for the assessment of the influence of the weak interactions. The results of the measurements of the neutrons’ population with the absorber are shown in Fig. 3. It is seen that for the spin-analysis of the state ${|{+x}\rangle }$, i.e., for the post-selection of ${|{\Psi ^{+}_f}\rangle }$, the interferogram is unchanged with the absorber in path I and only an absorber put in the path II has an influence. Nonetheless, for the spin-analysis of the state ${|{-x}\rangle }$, i.e., for the post-selection of ${|{\Psi ^{-}_f}\rangle }$, the position of the neutrons is swapped from the path II to the path I. By changing the state of the spin-analysis to ${|{-x}\rangle }$, the obtained results confirm that the position of the neutrons are now found in path I, while no effect is seen by putting the absorber in the other one, namely path II. It is concluded from the above results, that the position of the neutrons’ location depends solely on the choice of the post-selection, that is either ${|{\Psi ^{+}_f}\rangle }$ or ${|{\Psi ^{-}_f}\rangle }$.

**Figure 4:**
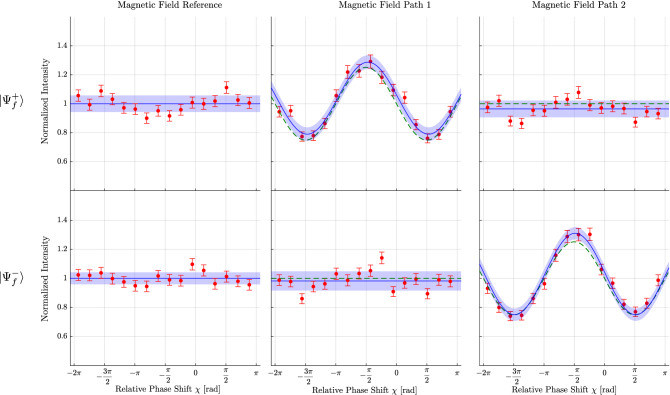
Typical result of the measurement of the location of the spin component Interferograms are recorded in the same manner as in Fig. 3. Three plots, one reference without the additional magnetic field and two with the magnetic field in the paths I/II, are obtained, each for the choices of the final states ${|{\Psi ^+_f}\rangle }$ and ${|{\Psi ^-_f}\rangle }$. The rotation angles by the additional magnetic field in the path I/II are set respectively to $\pm \pi /9$. The solid lines, error bars and the filled area represent the same as in Fig. 3. In comparison with the reference measurement on the left column, two features are observed. (1) For the post-selection of the state ${|{\Psi ^+_f}\rangle }$, the magnetic field in path I causes a sinusoidal modulation of the intensity and leaves it unchanged in path II; the spin-component traverses through the interferometer taking only path I. (2) For the post-selection of the state ${|{\Psi ^-_f}\rangle }$, the magnetic field in path I leaves the intensity unchanged, while in path II it causes a sinusoidal modulation; hence the spin-component traverses through the interferometer taking only path II. Here again, by switching the states of the post-selection, the position of the spin component is swapped between path I and path II.

Secondly, the location of the spin is evaluated by the use of the interaction with a weak magnetic field. For this purpose each of the spin-rotators in the interferometer is used. In addition to the $\pm \pi /2$ spin-rotation needed for the generation of the pre-selection state, a small extra spin rotation along the z-axis is induced. In particular the rotations of $\frac{\pi }{9}$ in path I and $- \frac{\pi }{9}$ in path II are applied, thus enabling a weak interaction of the neutrons with a magnetic field. Again a measurement without this additional magnetic field served as reference and the fast switch of the spin-analysis was turned on during the whole measurements. Similar to the weak absorber measurements, six interferograms were obtained: one without the magnetic field and one with the field turned on for path I respectively path II, and each of these three for both spin-analysis directions ${|{+x}\rangle }$ or ${|{-x}\rangle }$. Figure 4 depicts the results of the measurements of the spin’s position. One finds that, in comparison with the Fig. 3, the whole behavior is inverted. Here the interaction with the magnetic field has an influence in path $\text {I}$ only for the spin analysis of the ${|{+ x}\rangle }$ states, i.e., for the post-selection of ${|{\Psi ^{+}_f}\rangle }$ and the magnetic interaction shows intensity modulation in path $\text {II}$ only for the spin analysis of the ${|{- x}\rangle }$ states, i.e., for the post-selection of ${|{\Psi ^{-}_f}\rangle }$. These results indicate, that the spin is only located in one of the beam path of the interferometer and that this position changes accordingly with the choice for the post-selection.

**Figure 5:**
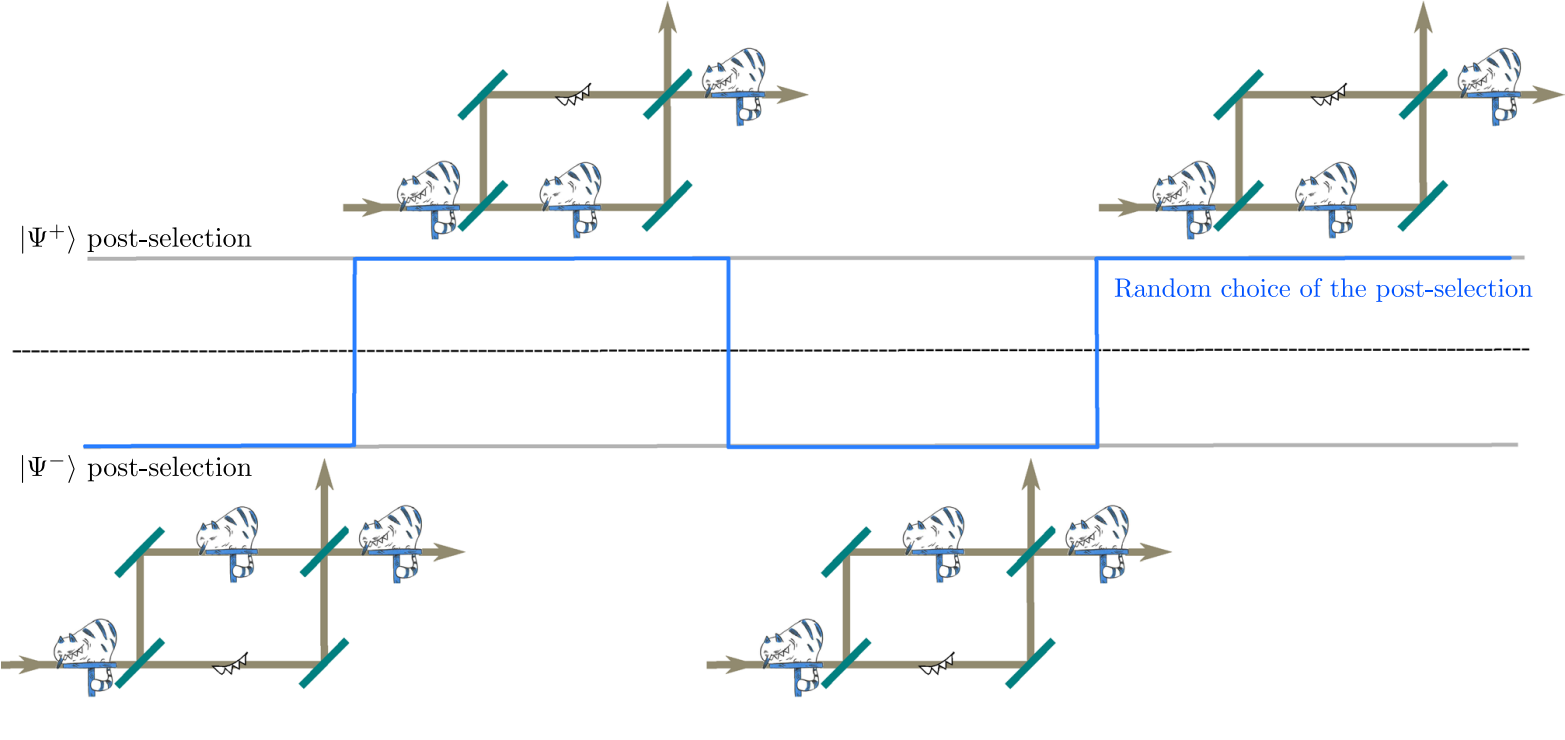
Graphical depiction of the emergence of the quantum Cheshire cat. The choice of the post-selection (blue) alternates randomly between ${|{\Psi ^{\pm }}\rangle }$. Although the cat (neutron) is not aware of these selection as it enters the interferometer, the locations of the cat and the grin are interchanged according to the delayed and random choice of the post-selection. Cat courtesy of Nicolas Mahler.

The results of the measurements above as a whole suggest that the location of the neutron and the position of the effective spin are found in different beam paths of the interferometer for the post-selections ${|{\Psi ^{\pm }_f}\rangle }$. The quantum Cheshire Cat emerges; the positions of the cat and the grin are interchanged according to the choice of the post-selection. A graphical depiction of this result, in particular for the post-selections ${|{\Psi ^{\pm }_f}\rangle }$, is shown in Fig. 5. Note that the choice of the post-selection changes randomly over time. That is, the choice of the post-selection is not decided at the time of the neutron’s entry into the interferometer and the consequences of the post-selection, i.e., the positions of the cat and grin are constituted afterwards at the time the post-selection is applied.

### Determination of the weak values

The interferograms, obtained by applying the weak absorber and the weak magnetic field interaction in combination with the fast switching of the the applied post-selections, allow us to make quantitative analysis of the behavior of the delayed choice qCC by determining the weak values. We used $\left\langle {\hat{\Pi }}_{{j}}\right\rangle ^\pm _{\mathrm{w}}$ for the analysis of the neutrons’ population and $\left\langle {\hat{\sigma }}_z{\hat{\Pi }}_{{j}}\right\rangle ^\pm _{\mathrm{w}}$ for the spins’ position, for each path, $j=\text {I, II}$ (superscript ± denotes the two different choices of the post-selection). For the evaluation of the neutrons’ population, the intensities $I^\pm _{ref}$ and $I^\pm _{abs-\text {I/II}}$ are used directly to determine the weak values $\left\langle {\hat{\Pi }}_{{j}}\right\rangle ^\pm _{\mathrm{w}}$ , where the super- and the sub-script denote again the two different directions of the spin analysis and the measurements without (as a reference)/with absorber in the path I/II, respectively. For the evolution of the spin’s position, the amplitudes of the intensity modulations in the interferograms are used to determine the weak values $\left| \left\langle {\hat{\sigma }}_z{\hat{\Pi }}_{{j}}\right\rangle ^\pm _\mathrm{w}\right|$. The intensity $I(\chi )^\pm _{mag-\text {I/II}}$ modulates with varying relative phase shift $\chi$. Together with the reference intensity $I(\chi )^\pm _{ref}$ the weak values are evaluated. Here again the super- and the sub-script denote two different choices of the post-selection and the measurements with the magnetic field in the path I/II, respectively. A detailed description of the data analysis and evaluation is presented in the *Methods* section. The calculated weak values are summarized in table 1. The error bars represent inaccuracies of the experiments and are caused by uncertainties in the state preparation due to finite degree of initial polarization, a depolarization caused by the absorber and misalignment of the spin manipulation. These effects lead to imperfect separation of the particle and its property and cause a deviation of the weak values from the theoretical predicted outcomes 0 and 1. The predictions are confirmed within the error of the measurement: neutrons and spin are found to be located in differing paths of the interferometer and their locations are solely determined on the choice of the post-selection, that has been either ${|{\Psi ^{+}_f}\rangle }$ or ${|{\Psi ^{-}_f}\rangle }$.

**Table 1:** Weak values retrieved from the delayed-choice qCC experiment. The lower columns show the theoretical predictions for comparison.

		${|{\Psi ^{+}_{f}}\rangle }$		${|{\Psi ^{-}_{f}}\rangle }$	
	Path	Absorber	Magnet	Absorber	Magnet
	*j*	$\left\langle {\hat{\Pi }}_{{j}}\right\rangle _{\mathrm{w}}^+$	$\left| \left\langle {\hat{\sigma }}_z{\hat{\Pi }}_{{j}}\right\rangle _\mathrm{w}^+\right|$	$\left\langle {\hat{\Pi }}_{{j}}\right\rangle _{\mathrm{w}}^-$	$\left| \left\langle {\hat{\sigma }}_z{\hat{\Pi }}_{{j}}\right\rangle _\mathrm{w}^-\right|$
Measurement	I	$0.096\pm 0.099$	$0.99\pm 0.12$	$0.989\pm 0.16$	$0.25\pm 0.08$
	II	$1.04\pm 0.17$	$0.26\pm 0.06$	$0.24\pm 0.103$	$1.12\pm 0.13$
Theory	I	0	1	1	0
	II	1	0	0	1

## Discussion

The qCC effect, i.e. the spatial separation of the property of a quantum system, is described in quantum mechanics with the formalism of pre- and post-selected ensembles^42^. This is in our case the spatial separation of the neutron’s spin from the particle’s localization in the interferometer and follows from observations of the effective consequences of absorbers and magnetic fields in the interferometer paths that probe either for the location or the property. In the present work, we considered the influence of the post-selection on the quantum Cheshire Cat; in particular an influence of a delayed-choice that lies in the future with regard to a point in time when the neutrons have already entered the interferometer. The central point of quantum theory, as Wheeler put it into one sentence: “No elementary phenomenon is a phenomenon until it is a registered (observed) phenomenon.”^10^, is demonstrated by the observed results of the presented experiment. The dynamical behavior was undefined even though the interaction with the probe system had already taken place.

The emergence of the quantum Cheshire cat with interchanging the positions of the cat and the grin according to the choice of the post-selection is depicted graphically in Fig. 5. It is worth noting here that not only the post-selections of ${|{\Psi ^{\pm }_f}\rangle }$ but also selections of some intermediate states, i.e. ${|{+z}\rangle }$ or any other, had been carried out in our experiment as well. For these intermediate states, no separate localization of neutron and spin-component is expected; the neutron and the spin-component would be distributed (in equal or unequal but non-vanishing parts) in both paths of the interferometer. The effect of disembodiment and the interchange of the localized properties in the interferometer is expected to be seen only for the combination of the post-selections of ${|{\Psi ^{\pm }_f}\rangle }$. Since the goal of the present experiment is to confirm the dynamical switching of the position and the grin of the quantum Cheshire cat according to the change of the post-selection, we have purposely selected and presented here only the results for the according post-selections.

The obtained results of the experiment confirm the fact that the location of the neutron and its spin can be interchanged actively for a suitable selected pairs of post-selections. This is presumed to be a consequence of quantum causality; the dynamical behavior of a quantum system is undefined until it is actually registered. Further investigations are required to answer the question, whether this dynamical consequence is attributed to the intrinsic indeterminacy of quantum mechanics^43^ or constitutes an influence of retro-causal origin.

## Methods

### Weak measurement of the neutrons’ location

To make the weak measurement for the neutrons’ location $\left\langle {\hat{\Pi }}_{\text {I}}\right\rangle _{\mathrm{w}}^\pm$ and $\left\langle {\hat{\Pi }}_{\text {II}}\right\rangle _{\mathrm{w}}^\pm$ (superscripts ${\pm }$ indicating the analysis direction), absorbers with a high transmissivity, in our experiment thin slabs of Indium, were put alternately into each path. This allows us to realize a “weak absorption”. The absorption can be represented as an imaginary optical potential with an absorption coefficient $\text {M}_j$ for path *j*^26^. In case the absorption is weak the transmissivity $T_{j}$ is related to $\text {M}_{j}$ through $\text {M}_{j}\approx 1-\sqrt{T_{j}}$. For small $\text {M}_{j}$ the wave function behind the absorber can be expressed as:

4 $$\begin{aligned} \left| \psi '\right\rangle = \mathrm e^{-M_{j}\hat{\Pi }_{j}} \left| \psi _{\mathrm{i}}\right\rangle \approx \left[1-\text {M}_{j}\hat{\Pi }_{j}+\cdots \right]\left| \psi _\mathrm{i}\right\rangle . \end{aligned}$$

Using the definition of the weak value, $\left\langle {\hat{\Pi }}_{j}\right\rangle _{w}^\pm =\frac{\langle \psi _\mathrm{f}^\pm |{\hat{\Pi }}_{j}|\psi _{\mathrm{i}}\rangle }{\langle \psi _\mathrm{f}^\pm |\psi _{\mathrm{i}}\rangle }$ the intensity for the post-selected outcome takes the form

5 $$\begin{aligned} I_{abs-j}^{\pm }=\left| \left\langle \psi _{\mathrm{f}}^\pm |\psi _\mathrm{i}\right\rangle \right| ^{2}\left[1-2\text {M}_j\left\langle {\hat{\Pi }}_{{j}}\right\rangle _\mathrm{w}^\pm \right]. \end{aligned}$$

From $I_{abs-j}^{\pm }$, together with the measured reference intensity without absorbers ${I}^{\pm }_{ref}=\left| \left\langle \psi _{\mathrm{f}}^\pm |\psi _\mathrm{i}\right\rangle \right| ^{2}$ and $\text {M}_{j}$ the weak values can be extracted via:

6 $$\begin{aligned} \left\langle {\hat{\Pi }}_{{j}}\right\rangle _{\mathrm{w}}^\pm = \frac{1+I_{abs-j}^{\pm }/ I^{\pm }_{ref}}{2 \text {M}_{j}}. \end{aligned}$$

### Weak measurement of the location of the spin component

To measure $\left\langle {\hat{\sigma }}_z{\hat{\Pi }}_{{j}}\right\rangle _{\mathrm{w}}^\pm$ the location of the spin component, a small magnetic field is applied in path *j*, inducing a path dependent spin rotation. The interaction Hamiltonian for this measurement is

7 $$\begin{aligned} {\hat{H}}_{j}=-\gamma \frac{{\hat{\sigma }}_z}{2}B_z{\hat{\Pi }}_j \end{aligned}$$

where $\gamma$ is the gyromagnetic ratio, $B_z$ the applied magnetic field and ${\hat{\Pi }}_{j}$ the denotation that the magnetic field is only applied along path *j*. Since the magnetic field is applied along ${\hat{z}}$, only the ${\hat{\sigma }}_{z}$ component of the Pauli matrix appears in the Hamiltonian. This generates a rotation $\alpha$ around the z-axis, by Larmor precession with its magnitude proportional to the magnetic field strength^44^. By choosing $\alpha$ to be small we guarantee a weak measurement. The evolution of the initial state caused by the weak measurement is given by

8 $$\begin{aligned} \left| \psi '\right\rangle =\mathrm e^{-\mathrm{i}\int \mathrm dt {\hat{H}}_{j}}\left| \psi _{\mathrm{i}}\right\rangle =\mathrm e^{\mathrm{i} \alpha {\hat{\sigma }}_z{\hat{\Pi }}_{{j}}/2}\left| \psi _\mathrm{i}\right\rangle \approx \left[1+\frac{\mathrm{i}\alpha }{2}{\hat{\sigma }}_z{\hat{\Pi }}_{{j}} - \frac{\alpha ^2}{8}{\hat{\Pi }}_{{j}} + \cdots \right]\left| \psi _{\mathrm{i}}\right\rangle . \end{aligned}$$

After post-selection for the outcomes corresponding to the final state $\left| \psi _{\mathrm{f}}\right\rangle$, the intensity for a magnetic interaction in path *j* at the O-detector is, taking into account $\alpha$ up to order ${{O}}(\alpha ^2)$,

9 $$\begin{aligned} \begin{aligned} {I}_{mag-j}^{\pm }&=|\langle \psi _{\mathrm{f}}^\pm |\psi '\rangle |^2 \\&\approx |\langle \psi _{\mathrm{f}}^\pm |\psi _{\mathrm{i}}\rangle |^2 -\frac{\alpha ^2}{4}\langle \psi _\mathrm{f}^\pm |{\hat{\Pi }}_{{j}}|\psi _{\mathrm{i}}\rangle \langle \psi _\mathrm{i}|\psi _{\mathrm{f}}^\pm \rangle +\alpha {\text {Im}} \Big [ \langle \psi _{\mathrm{f}}^\pm |{\hat{\sigma }}_z{\hat{\Pi }}_{{j}}|\psi _{\mathrm{i}} \rangle \langle \psi _{\mathrm{i}}|\psi _{\mathrm{f}}^\pm \rangle \Big ] +\frac{\alpha ^2}{4}|\langle \psi _\mathrm{f}^\pm |{\hat{\sigma }}_z{\hat{\Pi }}_{{j}}|\psi _{\mathrm{i}}\rangle |^2 . \end{aligned} \end{aligned}$$

Again using $\left\langle {\hat{\Pi }}_{j}\right\rangle _{w}^\pm =\frac{\langle \psi _\mathrm{f}^\pm |{\hat{\Pi }}_{j}|\psi _{\mathrm{i}}\rangle }{\langle \psi _\mathrm{f}^\pm |\psi _{\mathrm{i}}\rangle }$, and $\left\langle {\hat{\sigma }}_z{\hat{\Pi }}_{{j}}\right\rangle _{w}^\pm =\frac{\langle \psi _\mathrm{f}^\pm |{\hat{\sigma }}_z{\hat{\Pi }}_{{j}}|\psi _\mathrm{i}\rangle }{\langle \psi _{\mathrm{f}}^\pm |\psi _{\mathrm{i}}\rangle }$, we arrive at:

10 $$\begin{aligned} {I}_{mag-j}^{\pm }= \left| \left\langle \psi _{\mathrm{f}}^\pm |\psi _\mathrm{i}\right\rangle \right| ^2 \left[1 - \frac{\alpha ^2}{4}\left\langle {\hat{\Pi }}_{{j}}\right\rangle _\mathrm{w}^\pm - \alpha {\text {Im}} \Big [ \left\langle {\hat{\sigma }}_z{\hat{\Pi }}_{{j}}\right\rangle _{\mathrm{w}}^\pm \Big ] +\frac{\alpha ^2}{4}\left| \left\langle {\hat{\sigma }}_z{\hat{\Pi }}_{{j}}\right\rangle _\mathrm{w}^\pm \right| ^2\right]. \end{aligned}$$

We note that for $\left\langle {\hat{\sigma }}_z{\hat{\Pi }}_{{j}}\right\rangle _{\mathrm{w}}^\pm$ with our pre-and post-selected states Eq. (1) the general statement:

11 $$\begin{aligned} \left\langle {\hat{\sigma }}_z{\hat{\Pi }}_{{j}}\right\rangle _{\mathrm{w}}^{\pm } = \mathrm e^{\pm \mathrm{i} \chi } \end{aligned}$$

holds. Hence

12 $$\begin{aligned} {\text {Im}} \Big [ \left\langle {\hat{\sigma }}_z{\hat{\Pi }}_{{j}}\right\rangle _{\mathrm{w}}^{\pm } \Big ] = \pm \sin (\chi ) \end{aligned}$$

To take into account experimental imprecisions in the preparation the modulus of the weak value may deviate from 1 so we get a general expression (see^27^):

13 $$\begin{aligned} {\text {Im}} \Big [ \left\langle {\hat{\sigma }}_z{\hat{\Pi }}_{{j}}\right\rangle _{\mathrm{w}}^{\pm } \Big ] = \pm \sin (\chi ) \left| \left\langle {\hat{\sigma }}_z{\hat{\Pi }}_{{j}}\right\rangle _\mathrm{w}^{\pm }\right| \end{aligned}$$

So Eq. (10) becomes:

14 $$\begin{aligned} {I}_{mag-j}^{\pm }= \left| \left\langle \psi _{\mathrm{f}}^\pm \psi _\mathrm{i}\right\rangle \right| ^2 \left[1 - \frac{\alpha ^2}{4}\left\langle {\hat{\Pi }}_{{j}}\right\rangle _{\mathrm{w}} \mp \alpha \sin (\chi ) \left| \left\langle {\hat{\sigma }}_z{\hat{\Pi }}_{{j}}\right\rangle _\mathrm{w}^{\pm }\right| +\frac{\alpha ^2}{4}\left| \left\langle {\hat{\sigma }}_z{\hat{\Pi }}_{{j}}\right\rangle _\mathrm{w}\right| ^2\right]. \end{aligned}$$

Hence we can retrieve the weak value from the amplitudes of a sine fit to the intensities and normalize with ${I}^{\pm }_{ref}=\left| \left\langle \psi _{\mathrm{f}}^\pm |\psi _\mathrm{i}\right\rangle \right| ^{2}$, the reference intensity without magnetic interaction.

### Additional details on data treatment

The pre- and post-selected states ${|{\Psi _{i}}\rangle }$ and ${|{\Psi ^{\pm }_{f}}\rangle }$ for our experiment are given by Eq. (1). With the interaction according to Eq. (9) the expected intensities can be calculated (see^26^). We get for ${|{\Psi ^{+}_{f}}\rangle }$ and interaction in path I:

15 $$\begin{aligned} \begin{aligned} I^{mag~+}_{out-\text {I}}&= \frac{1}{8}\Big (3 - 4\sin (\frac{\alpha }{2})\sin (\chi ) - \cos (\alpha )\Big ), \end{aligned} \end{aligned}$$

while the expression for interaction in path II is:

16 $$\begin{aligned} \begin{aligned} I^{mag~+}_{out~\text {II}}&= \frac{1}{4}\cos ^2(\frac{\alpha }{2}) \end{aligned} \end{aligned}$$

For analysis direction ${|{\Psi ^{-}_{f}}\rangle }$ we get expressions that are analogous to the two equations above, but with behavior inverted for the two paths. So the dependency on $\chi$ is retrieved for interaction in path II and the $\chi$-independent intensity is retrieved for path I. These expressions can now be normalized with the reference intensities and further simplified by expanding the expressions around $\alpha$ and ignore terms of the order ${O}(\alpha ^2)$ or higher (weak regime). The intensities for both analysis directions are:

**Post-selection**  $${|{\Psi ^{+}_{f}}\rangle }$$

17 $$\begin{aligned} I^{mag~+}_{norm~\text {I}}&= I^{mag~+}_{out~\text {I}}\Big /{I^{+}_{ref}} = 1 - \alpha \sin (\chi ) \end{aligned}$$

18 $$\begin{aligned} I^{mag~+}_{norm~\text {II}}&= {I^{mag~+}_{out~\text {II}}} \Big / {{I^{+}_{ref}}} = 1 \end{aligned}$$

**Post-selection**  $${|{\Psi ^{-}_{f}}\rangle }$$

19 $$\begin{aligned} I^{mag~-}_{norm~\text {I}} = {I^{mag~-}_{out~\text {I}}} \Big / {I^{-}_{ref}}&= 1 \end{aligned}$$

20 $$\begin{aligned} I^{mag~-}_{norm~\text {II}} = I^{mag~-}_{out~\text {II}} \Big / I^{-}_{ref}&= 1 + \alpha \sin (\chi ) \end{aligned}$$

In addition a reference interferograms $I^{ifg}_{ref}$ with the same phase shifter positions have been recorded with the state preparation and the delayed choice coils turned off. These interferograms are used to determine the contrast of the interferometer and to define the $\chi$ positions of the phase shifter.

For the curves of the theoretical prediction of the effect of the spin rotation, the finite contrast was taken into account. The value of the mean contrast during our measurement campaign was $C = 0.72$ and has been retrieved from fits to reference interferograms $I^{ifg}_{ref}$ with the state preparation and the delayed-choice coils turned off. The expected intensities are then:

21 $$\begin{aligned} I^{mag~\pm }_{rot~\alpha } = 1 \mp C\alpha sin(\chi ). \end{aligned}$$

From the equation above and Eq. (14) we see that via $\left| \left\langle {\hat{\sigma }}_z{\hat{\Pi }}_{{j}}\right\rangle _\mathrm{w}^{\pm }\right| = {A} / {(\alpha C)}$, with *A* being the normalized amplitude of the sine fit to the intensities, the weak values can be calculated. For the absorber measurements the normalized intensities are independent from the phase shifter position $\chi$. The expected intensities, following from Eq. (5), are:

**Post-selection**  $${|{\Psi ^{+}_{f}}\rangle }$$

22 $$\begin{aligned} I^{abs~+}_{norm~{\text {I}}}&= I^{abs~+}_{out~\text {I}}\Big /{I^{+}_{ref}} = 1 \end{aligned}$$

23 $$\begin{aligned} I^{abs~+}_{norm~{\text {II}}}&= {I^{abs~+}_{out~\text {II}}} \Big / {{I^{+}_{ref}}} = 1 - 2\big (1-\sqrt{T_{Indium}}\big ) \end{aligned}$$

**Post-selection**  $${|{\Psi ^{-}_{f}}\rangle }$$

24 $$\begin{aligned} I^{abs~-}_{norm~{\text {I}}}&= I^{abs~-}_{out~\text {I}}\Big /{I^{-}_{ref}} = 1 - 2\big (1-\sqrt{T_{Indium}}\big ) \end{aligned}$$

25 $$\begin{aligned} I^{abs~-}_{norm~{\text {II}}}&= {I^{abs~-}_{out~\text {II}}} \Big / {{I^{-}_{ref}}} = 1 \end{aligned}$$

The experimental results are in good agreement with the theoretical calculations (Fig. 4). These reference interferograms have been used to fix $\chi$ positions of the phase shifter. The weak values (Tab.1) have been for calculated from intensity values, that were retrieved from the fits, at position $\chi =0$. The errors quoted of the weak values are from propagation of the standard deviations of the counting statistics and the systematic effects due to uncertainties in the absorber thickness and the adjustment of the rotation angle $\alpha$. For 16 different phase shifter positions (in the range ${-2\pi }$ to ${\pi }$ with a step size of ${0.59}\hbox {rad}$), a 5 minute delayed- choice measurement has been recorded. For each given configuration, the 16 delayed-choice measurements are grouped together to form a full interferogram. In addition a reference interferograms $I^{ifg}_{ref}$ with the same phase shifter positions have been recorded with the state preparation and the delayed-choice coils turned off. These interferograms are used to determine the contrast of the interferometer and to define the $\chi$ positions of the phase shifter.

### Experimental technique for the fast switch of the delayed-choice option

The *O*-beam was recorded with a $^{3}$He pencil-sized detector, *E68932* from TOSHIBA. The arrival time of each neutron is recorded. This is necessary to reconstruct the switching state of the delayed-choice coil (ST2). The distance from the first interferometer plate to the ST2 is $\sim 35$ cm. The neutrons have a velocity of ${2060}\,\hbox {ms}^{-1}$. To guarantee, that the state is completely random and uncorrelated to the time the neutron has entered the interferometer, we have to make sure that a decision or choice to switch the analysis direction is made at least once every ${170}\,{\upmu \hbox {s}}$. Our random signal is generated with the help of a Raspberry Pi, *RPi 2 Model B* We use the hardware random number generator (HWRNG) functionality of the Pi’s system on chip (SoC). With the *rng-tools* package it is possible to generate true random numbers. We read a random bit of the *Raspberry Pi* every ${50}\,{\upmu \hbox {s}}$ and switch the voltage of a defined output pin (11) between high and low according to the random bit. The software to perform this task on the *Raspberry Pi* is a script written in the programming language Python^45^, that uses the wiringPi framework to set the the Pi’s GPIO-pin 11 to a voltage of ${3.3}\,\hbox {V}$ for random bit being 1 and ${0}\,\hbox {V}$ for random bit being 0. This random signal is transmitted by wiring the GPIO pin of the *Raspberry Pi* to the digital input pin of a FPGA board (NI PXI-7833R, National Instruments). On the FPGA card a LabVIEW (National Instruments) program reads the random, logical state of the incoming signal every ${100}\,{\upmu \hbox {s}}$ and sends the according $+\pi /2$ voltage (${|{\Psi ^+_f}\rangle }$ post-selection) for state being one respectively the $-\pi /2$ voltage (${|{\Psi ^-_f}\rangle }$ post-selection) for state being zero to the ST2 coil. The reason for this down-sampling strategy is, that the *Raspberry Pi* has no real-time functionality, whereas the FPGA board in contrast does. So drifts in the loop time of the random bit signal from the *Raspberry Pi* are taken care off. The LabVIEW program on the FPGA board handles further the time resolved measurement of the incoming neutrons (single events) and combines the TTL signal of neutron detectors with the random numbers from the *Raspberry Pi* so that in retrospect the switching state of the ST2 coil can be assigned to the detection times of the neutrons.

**Figure 6:**
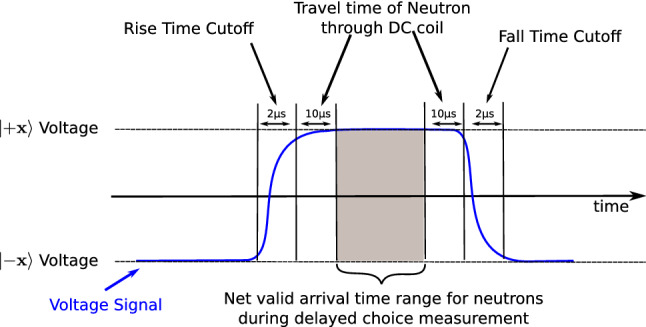
Timing considerations for the delayed choice switching between ${|{\pm x}\rangle }$ voltages, corresponding to the $|\Psi ^\pm _f\rangle$ post-selections. The current in the ST2 (see Fig. 2) coil has to be constant while the neutrons pass it. The rise time (resp. fall time) from one current level to the other and the neutron travel time through the ST2 coil define periods which cannot be used for the experiment. Only neutrons with specific arrival times in the grey area can be used for the measurement. To achieve this the arrival times and the flip state of the ST2 coil had to be recorded and matched with the voltage state of the ST2 at the moment the neutron was passing. Trimming the shape of the rise and fall of the signal, to make them steeper, was accomplished by putting additional resistors in series with the ST2 coil.

The fast switching of the current of the delayed-choice spin rotator ST2 was achieved with the 4-quadrant DC amplifier *TOE 7610-20* from *TOELLNER*, that could provide up to 150 W of source and sink power. The ST2 component had an inductivity of $L = {12.2}\,{\upmu {\hbox {H}}}$. The current necessary for a $+\pi /2$ or $-\pi /2$ flip was $I=\pm {2.25}{A}$. To obtain a good shape of the switching signal, ideally a perfect square wave, the rise time (defined by $\tau =L/R$^46^) was decreased, by putting a resistor in series with the coil (total $R = {6.1}\Omega$), to yield a $\tau$ of ${2}\,{\upmu \hbox {s}}$. For the detected neutron event, one has to take into account the voltage state of the ST2 coil, at the moment of the passing by of the neutron. A neutron can only be used for the measurement, if the voltage of the coil was constant for the time the neutron spend passing through the region of the coil. This flight through time is roughly ${10}\,{\upmu \hbox {s}}$ for neutrons with velocity of about ${2000}\,\hbox {ms}^{-1}$ and a coil width of 20 mm. Another important issue to take into consideration is the rise and fall time of the voltage signal when the level changes from $+\pi /2$ to $-\pi /2$ spin rotation currents and vice versa due to the inductance of the coil. For each detected neutron one of the following decision is made: a) if the neutron is detected and the voltage level is high ($+\pi /2$ spin rotation), the event is used for ${|{\Psi ^+_f}\rangle }$ post-selection analysis b) if the neutron is detected and the voltage level is low ($-\pi /2$ spin rotation), the event is used for ${|{\Psi ^-_f}\rangle }$ post-selection analysis c) if the neutron is detected during voltage the transition periods from high to low (respectively from low to high), the event is dropped for the analysis. In Fig. 6 the situation is schematically depicted. The grey shaded area indicates the time where the neutron can be included to the results. To achieve this classification, the arrival times and the flip state of the ST2 coil had to be recorded and matched. This has been done as a post-processing data analysis step.

## Data Availability

The data that was analysed for the findings of this report is available under https://doi.ill.fr/10.5291/ILL-DATA.3-16-1.

## Code availability

The following software packages used for the experiment are freely available online: rng-tools:https://github.com/nhorman/rng-tools wiringPi:http://wiringpi.com.
